# Appropriate Regulation of GM Insects

**DOI:** 10.1371/journal.pntd.0001496

**Published:** 2012-01-31

**Authors:** Luke Alphey, Camilla Beech

**Affiliations:** 1 Oxitec Limited, Oxford, United Kingdom; 2 Department of Zoology, University of Oxford, Oxford, United Kingdom; Liverpool School of Tropical Medicine, United Kingdom

After many years of open discussion and development [1]–[8], the first genetically modified (GM) insect strains are entering field trials [9], [10]. A key engineered trait renders the insects “genetically sterile”, such that some or all of their offspring die [11]–[14]; the insects additionally carry a fluorescent marker gene for easy identification. Such “genetic sterility” transgenes (in genetic terms, conditional dominant lethal genes) are not able to establish or spread in the wild due to their high fitness cost; such self-limiting strategies are widely viewed as the lowest risk category (e.g., [15]). Other genetic strategies are in development, including more invasive genetic systems. Some of these, such as those based on artificial infection with *Wolbachia* [16], may not be covered by regulations narrowly focused on the use of recombinant DNA technology despite having many similar properties [17].

In their provocative opinion piece, Reeves et al. question aspects of the regulatory process for GM insects [18]. Approval for limited field releases of GM insects has been given in four countries at the time of writing, and Reeves et al. consider aspects of three of these. Their exclusive focus on GM insects allows room for some interesting speculation about some potential features of an idealised regulatory system. Conversely, ignoring established regulatory systems and experience in other areas such insecticides, drugs, vaccines, and GM crops unfortunately leads them to make unworkable recommendations in several areas.

Regulatory agencies seek to permit the development of beneficial new technology while minimising any potential harms. Regulatory systems therefore need to identify and characterise potential harms, and determine whether these should be accepted, mitigated, or avoided. The degree of uncertainty in any a priori analysis, especially where new technologies are concerned, leads to an element of judgement, as does the identification and weight attached to various protection goals. In some cases, potential benefits may be taken into account and weighed against potential risk. One indirect approach to this is to compare proposed actions with the “no action” alternative or status quo. For insect control, this is the current use of chemical insecticides plus uncontrolled damage from the insect, e.g., for the dengue mosquito *Aedes aegypti* 50–100 million cases of dengue per year.

Reeves et al. correctly note the need for a science-based, case-by-case assessment. For example, the ecological impact of suppressing an invasive insect pest may be very different in its region of origin relative to an area where it is has recently established. However, the authors then criticise the United States government's regulation of GM insects on the ground that one component, an environmental impact statement (EIS) [19], was not sufficiently case-specific. This criticism is misplaced. As Reeves et al. note elsewhere, “EIS documents can have a very broad scope, as they are principally intended to evaluate the impact of proposed agency policy changes on a broad programmatic basis at a national level. EA [Environmental Assessment] documents instead are generally focused on specific actions in a single species at named locations”. The US Department of Agriculture (USDA) is to be commended on developing an EIS under the National Environmental Protection Act to assess potential future programmatic use of GM insects to augment its existing sterile-insect control programmes. In addition, specific proposed activities with engineered pink bollworm (*Pectinophora gossypiella*, a pest of cotton) were given case-by-case assessment under the Plant Protection Act. Remarkably, despite the longer history, rapid adoption, and large-scale use of GM crops, this was the first EIS completed for any genetically modified organism. This highlights the proactive stance taken by US regulatory agencies with respect to GM insects, as both developers and sceptics such as Reeves et al. would doubtless wish.

Reeves et al. call for more transparency in the process, arguing that it would instill greater public confidence if the regulators were to “show their working” by publishing all permit applications, associated data and reasoning, and detailed justification of their conclusions. This argument has some merit, but needs to be balanced against significant practical difficulties. Technology developers have legitimate rights to protect proprietary information; governments understand this and provide statutory protections. Reeves et al. criticise these protections without any recognition of the reasons for their existence. Fortunately, this is not an issue new to genetically modified organisms, and one can look elsewhere for a balanced discussion, for example Walport and Brest [20]. These authors are from philanthropic agencies funding the research and strongly preferring full publication, yet still recognise the ensuing trade-offs and pitfalls; developers might look for stronger protection.

Reeves et al. confuse the concepts of transparency, independence, and scientific quality. Regulators make robust, science-based decisions, taking data and input from a wide range of sources and using internal and external multi-disciplinary experts to carefully assess data quality and gaps and potential harms and mitigations, and ultimately determine a course of action that appropriately protects all stakeholders. It is highly desirable that regulators operate and communicate their decisions and the background to them as fully as possible, but this is a separate issue from the scientific quality of the decisions themselves. Regulators are of course independent of the applicants, but it is nonsense to suggest that the data inputs themselves should be independent. Inevitably, the applicants will know more about the proposed action and specifics of their technology and strains than anyone else. Regulators demand comprehensive information from the applicants, while recognising that applicants may have legitimate needs for confidentiality. This requirement for full information to ensure rigorous quality is therefore somewhat at odds with the separate desire for transparency. Regulators are well used to handling this tension, and do so in accordance with their national culture and legislative framework. None of this is unique to GM insects, arising similarly in the regulation of drugs, vaccines, and even non-technology issues such as construction.

Reeves et al. are also on weak ground when they assert that regulators should consider only information published in peer-reviewed journals. This assertion depends on three assumptions: that journal peer-review is a superior guarantee of quality than any other method, that no data from any other source can be of adequate quality to warrant consideration, and that regulators themselves are incapable of adequately assessing the quality and significance of data provided to them. Each of these assumptions is naïve at best.

Experienced researchers know that journal peer-review is an imperfect process, a trade-off between economy of effort and rigour, and is highly variable. An editor and two or so reviewers with some relevant experience will review the paper, typically focusing on their own area of expertise. For comparison, the USDA's EIS had a public scoping period to incorporate the widest possible range of potential concerns from the outset, and a public comment period to solicit comments on the draft. Unlike journal peer-review, where competitors and others likely to have negative views are routinely excluded, the draft EIS was specifically sent to multiple GM-sceptic non-governmental organisations and individuals to solicit their comments, as well as to independent scientific experts and others; in total, over 50 individuals. Furthermore, the availability of the draft EIS was advertised on the Federal Register and public meetings held in Washington, D.C., and four representative states to allow yet more opportunity for comment; these public meetings included presentations by USDA scientists on the technology and the implications of its use. Journals do not do this for papers, not because it is an inferior process, quite the opposite, but simply because so comprehensive a system would be unmanageable and unaffordable.

Furthermore, journals select papers for publication using criteria that are not fully congruent with the needs of regulators. In particular, it is difficult to publish negative data. Studies showing lack of difference between a transgenic strain and its unmodified wild-type counterpart may be of great interest to regulators but not to journal editors. It took years to publish the USDA-Oxitec field trials of an engineered strain of pink bollworm, in part because editors at several journals felt that since the GM strain performed as predicted from prior lab studies, and showed no strange behaviour relative to a non-GM comparator strain, the paper was not sufficiently exciting for their journal. *PLoS ONE*, unusually, looks for quality irrespective of “novelty” and published the paper [9].

Reeves et al. display a striking distrust of the regulatory process, and indeed of regulators themselves, which distorts their analysis. The ultimate responsibility for decision-making in this area lies with sovereign states. All of the countries discussed by Reeves et al. have national legislation for environmental protection, animal welfare, disease mitigation and control, etc., which covers the field use of GM insects irrespective of whether the country has chosen to regulate by technology process. Such legislation will take into account obligations from international agreements to which they are party (e.g., Cartagena, North American Plant Protection Organization [NAPPO]). Indeed, a narrow focus on recombinant DNA technology has led to regulatory gaps in dealing with other genetic technologies such as *Wolbachia*, *cis*-genics, and in vivo site-directed mutagenesis. Independent regulatory agencies, established under national legislation, review the proposal and data provided by applicants. Such agencies have broad scientific expertise, which they supplement as needed by consulting external experts. This provides the broad and deep independent scientific review advocated by Reeves et al., though by a different mechanism. Both scoping and review can engage a large number of experts across a wide range of disciplines, as illustrated above for the US EIS. Reeves et al. rightly commend the Malaysian regulators for their extraordinary efforts to solicit comment both before and after approving a limited field release of a GM mosquito.

Though the checklist provided by Reeves et al. is completely inappropriate for its proposed purpose, assessing scientific quality, the general concept of checklists within the regulatory process is a good one. One step in the process is to identify the widest possible range of potential harms, which can then be assessed for likelihood and consequence in respect of the specific proposed action. Checklists can help with this, though inevitably many items on a comprehensive checklist of potential harms will not apply to a specific proposal. This approach informed the development of NAPPO standard RSPM 27 concerning transgenic arthropods, and has been adopted in one form or another by several subsequent initiatives. This may also help promote a harmonised approach that would inform decision-making and aid transparency across multiple nations and agencies. External comment at this scoping stage can also be very helpful, and was used by the US and Malaysian regulatory authorities, for example.

Attention often focuses on the scientific and technical aspects of new technology, rather than regulatory processes, so in that respect the Viewpoint from Reeves et al. is welcome. However, their specific prescriptions would harm, rather than help, these processes. Fortunately, regulators have a much broader perspective and experience than Reeves et al. provide, and have proven well able to meet the challenge of regulating GM insects, at least for the relatively simple, minimal-risk systems so far developed.

Regulations and regulatory processes need to be consistent and proportionate. This means that different genetic technologies should be subject to similar scrutiny and assessment irrespective of their methodological details—in other words, treated consistently. Regulatory requirements should also be proportionate to potential harm—as Reeves et al. note, the present GM insects are all in the lowest-risk “sterile-insect” category. Consistent and proportionate regulatory systems recognise this, and also that other genetic technologies designed to persist in the environment may present very different risk categories. So far, the agencies tasked to regulate GM insects have appropriately taken a cautious, thorough approach that allows progress towards realisation of the substantial benefits GM insect technology could potentially provide, while rigorously protecting the public and environment.
